# Potential of Autologous Adipose-Derived Mesenchymal Stem Cells in Peritoneal Fibrosis: A Pilot Study

**DOI:** 10.34172/aim.2023.16

**Published:** 2023-02-01

**Authors:** Amin Ahmadi, Reza Moghadasali, Iraj Najafi, Soroosh Shekarchian, Sudabeh Alatab

**Affiliations:** ^1^Department of Stem Cells and Developmental Biology, Cell Science Research Center, Royan Institute for Stem Cell Biology and Technology, ACECR, Tehran, Iran; ^2^Research Center for Pharmaceutical Nanotechnology, Biomedicine Institute, Tabriz University of Medical Sciences, Tabriz, Iran; ^3^Nephrology Research Center, Shariati Hospital, Tehran University of Medical sciences, Tehran, Iran; ^4^Maastricht University Medical Center, Maastricht, Limburg, Netherlands; ^5^Digestive Disease Research Center, Digestive Disease Research Institute, Tehran University of Medical Sciences, Tehran, Iran

**Keywords:** End stage renal disease, Mesenchymal stem cells, Peritoneal dialysis, Peritoneal fibrosis, Ultrafiltration failure

## Abstract

**Background:**

We aimed to determine the effects of systemic therapy with autologous adipose tissue derived mesenchymal stem cells (AD-MSCs) on different parameters of peritoneal function and inflammation in peritoneal dialysis (PD) patients.

**Methods:**

We enrolled nine PD patients with ultrafiltration failure (UFF). Patients received 1.2±0.1×10^6^ cell/kg of AD-MSCs via cubital vein and were then followed for six months at time points of baseline, 3, 6, 12, 16 and 24 weeks after infusion. UNI-PET was performed for assessment of peritoneal characteristics at baseline and weeks 12 and 24. Systemic and peritoneal levels of tumor necrosis factor α (TNF-α), *interleukin-6 (IL-6)*, IL-2 and CA125 (by ELISA) and gene expression levels of transforming growth factor beta (TGF-β), smooth muscle actin (𝛼-SMA) and fibroblast-specific protein-1 (FSP-1) in PD effluent derived cells (by quantitative real-time PCR) were measured at baseline and weeks 3, 6, 12, 16 and 24.

**Results:**

Slight improvement was observed in the following UF capacity indices: free water transport (FWT, 32%), ultrafiltration - small pore (UFSP, 18%), ultrafiltration total (UFT, 25%), osmotic conductance to glucose (OCG, 25%), D/P creatinine (0.75 to 0.70), and Dt/D0 glucose (0.23 to 0.26). There was a slight increase in systemic and peritoneal levels of CA125 and a slight decrease in gene expression levels of TGF-β, α-SMA and FSP-1 that was more prominent at week 12 and vanished by the end of the study.

**Conclusion:**

Our results for the first time showed the potential of MSCs for treatment of peritoneal damage in a clinical trial. Our results could be regarded as hypothesis suggestion and will need confirmation in future studies.

## Introduction

 Chronic kidney disease (CKD) is a slow, progressive and irreversible loss of kidney function. Based on the latest GBD report in 2017, 697.5 million subjects suffered from all-stage CKD worldwide.^1^ A study in Iran reported 8.9% of subjects with CKD stages III–V.^2^ Importantly, CKD may finally progress towards end-stage renal disease (ESRD), in which patients need kidney transplantation or dialysis to survive.

 Peritoneal dialysis (PD) is the healthiest renal replacement modality until kidney transplantation can be performed. In our country, continuous ambulatory peritoneal dialysis (CAPD) as the most common form of PD, comprises 4.1% of all renal replacement modalities.^3^ Despite the advantages of this modality, preserving the peritoneal membrane (PM) filtration capacity presents the most critical challenge in long-term CAPD patients. The process of fluid removal as well as solute clearance is done through the mechanism of peritoneal ultrafiltration (UF) in PD patients. If PD fails to remove the solute and fluid to match the needs of patients, they experience a situation called ultrafiltration failure (UFF) which is one of the most common causes of PD drop out.^3^ Peritoneal fibrosis (PF) is the main cause of filtration capacity loss that has two cooperative parts of fibrosis and inflammation with inflammation usually proceeding to fibrosis.^4^ Repeated episodes of peritonitis in addition to prolonged exposure of PM to bio-incompatible PD solutions trigger production of various cytokines mediating inflammatory, fibrogenesis and angiogenesis processes. The final ultimate product of initiation of this mechanism is progressive detachment of mesothelial cell layer as well as its transformation into fibroblastoid cells through a process called mesothelial-mesenchymal transition (MMT).^4^ Morphologically, the interaction of these two arms results in mesothelial cell loss, submesothelial compact zone thickening, abnormal angiogenesis and ultimately UFF.^5^ UFF is common in about 20% of patients undergoing long-term PD.^6^ Currently, the possibilities for modifying this process are limited and therefore, it is crucial to explore the therapeutic approaches to alleviate or inhibit PF.

 Over the last three decades, tremendous progress has been made in the field of regenerative medicine. Based on the properties of mesenchymal stem cells (MSCs),^7,8^ these cells have been evaluated in experimental studies for their therapeutic effects and researchers have started to translate these experimental studies into pioneering clinical trials.^9-11^ Regarding PD, we have shown in a systematic review that use of stem cells in experimental studies is associated with significant improvement in structural indices of PF as well as the functional capacity of PM.^12^ Based on these observations, in a previous pioneer pilot trial, we showed that autologous adipose tissue-derived MSCs (AD-MSCs) can be obtained, proliferated *in vitro* and injected safely via intravenous (IV) infusion to patients who were on maintenance CAPD.^13^ Here in this study, we aimed to further explore the effects of MSCs on PM characteristics. We evaluated the effects of AD-MSCs treatment on the expression levels of transforming growth factor beta (TGF-β), fibroblast-specific protein-1 (FSP-1) and smooth muscle actin (𝛼-SMA) on cells derived from PD-effluent. Studies showed that TGF-β is the main fibrogenic signal conductor for development of PF.^4,5^ In the normal peritoneum, the resident fibroblasts are scattered in the submesothelial connective tissue and identified by their spindle-shape appearance and expression of FSP-1. Following stimulation with fibrogenic factor TGF-𝛽, fibroblasts transform into an activated phenotype that is characterized by the expression of 𝛼-SMA.^5^ Evaluating the expression levels of these genes following MSCs treatment could help us to clarify the probable underlying mechanism of the effects of MSCs treatment on PF.

## Materials and Methods

###  Study Design and Patient Selection

 Many preclinical, clinical and meta-analysis studies have shown the safety of autologous MSCs in different animal models and populations.^14^ Moreover, preclinical studies of animal models of PD have shown safety of MSCs. Based on these observations and published data, we designed our study as a prospective, open-label, pilot study to evaluate the PM characteristics and cellular changes after single IV infusion of AD-MSCs in PD patients.

 The Institutional Review Board and the local Ethics Committee of Tehran University of Medical Sciences (code: 93-03-47-27290-146850) and Royan Institute (IR.ACECR.ROYAN.REC.1395.2294000123) approved this study. The IRCT of this study is IRCT2015052415841N2. All participants gave informed written consent. The complete protocol has been provided previously.^13^ As we have previously shown that UFF is a risk factor for developing severe PF,^15^ we included adult patients (aged > 18 years) who were on CAPD for at least 2 years and suffered from UFF. UFF was defined as UF < 400 mL after 4-hour dwell duration with 4.25% dextrose-based PD fluid. Patients needed to be free of peritonitis at the time of enrollment and follow-up sampling.

###  Isolation, Expansion of AD-MSCs

 The complete protocol of AD-MSCs isolation, expansion, immunophenotyping, and quality control has been reported previously.^13^ In brief, autologous adipose tissue was collected under local anesthesia through lipo-aspiration at Royan Institute. Samples were washed, enzymatically digested and centrifuged. The pellets were then filtered and plated in culture flask till 90% confluency was achieved. The washed and detached cells were counted and loaded into 10-mL sterile syringes for IV administration. The immunophenotypes of cells were characterized for each patient using 2 × 10^5^ MSCs from passage 2 cells.

###  Quality Control Tests of AD-MSCs

 For quality control, the following tests were performed: limulus amebocyte lysate gel clot assays, analysis of microbial tests, detection of mycoplasma and finally, karyotyping. All the procedures were done according to the protocols of the “Iranian Health Ministry Pharmacopoeia Commission” and “Department of Health and Human Services Food and Drug” for cell and tissue validation tests.^13^

###  AD-MSCs Administration

 AD-MSCs were suspended in 50 mL normal saline and injected under sterile conditions through the cubital vein according to our infusion protocol. Each patient received at least 10^6^ cells/kg. We chose this dose of AD-MSCs based on studies performed in animal models of PD as well as clinical trials of CKD patients.^9,12^ The complete details of safety assessment have been provided previously.^13^ In brief, safety was evaluated based on occurrence of serious and non-serious adverse events, and also the abnormalities in laboratory data according to the Common Terminology Criteria for Adverse Events.

###  Assessments of Peritoneal Membrane Function

 For assessment of peritoneal function, we used the UNI- peritoneal equilibration test (UNI-PET) before (V- 1) and 12 (V- 4) and 24 weeks (V- 6) after treatment with AD-MSCs. This test is a combination of ordinary 3.86%-PET and Double Mini-PET.^16^ In brief, after an 8–12-hour overnight dwell with 1.36% glucose solution with lactate as buffer, dialysate was drained and volume was measured. Then, a fresh 2 L 1.36% glucose solution was infused. Duration of infusion was recorded and fresh PD fluid samples were taken from the bags at the end of infusion. After 1 hour, complete drainage was done with the volume and duration recorded. A 5-mL blood sample and 10 mL of PD effluent were collected for laboratory measurements. The steps of infusion and drainage were repeated with 3.85% glucose solution. Then, the drained dialysate was flushed back into the peritoneum with the volume and duration recorded and samples collected. After 3 hours, complete drainage (by gravity in 20 minutes) was done with the volume and duration of infusion recorded and samples collected from the dialysate and blood. All collected samples from blood and PD effluent were sent to one specific laboratory for measuring the levels of blood urea nitrogen (BUN), creatinine, glucose, Na, albumin and total protein. The measured parameters were calculated according to La Milia.^16^

 Dt/D0 = dialysate glucose concentration (mg/dL) at the end of the test / “fresh” solution glucose concentration (mg/dL).

 D/P creatinine = dialysate-to-plasma creatinine (mg/dL) concentration ratio at 4 h

 D/P Urea = dialysate-to-plasma Urea (mg/dL) concentration ratio at 4 h

 Osmotic conductance to glucose (OCG) (mL/min/mm Hg) = {(V3.86 – V1.36)/ [19.3 × (G3.86 – G1.36) × t]} × 1.7

 Free water transport (FWT) (assessed during the second part of the test (3.86% glucose solution)) = ultrafiltration total (UFT) (mL) – ultrafiltration of small pore (UFSP) (mL)

 UFSP (assessed using Na clearance) = [NaR × 1,000]/Na plasma

 Na R (mmol) (Na removed during the second part of the test with the 3.86% solution) =


$$\begin{array}{l} \left[Volume_{DialysateOut}\left(L\right)Na_{DialysateOut}\left(mmol/l\right)\right]\\ -\left[Volume_{DialysateIn}\left(L\right)Na_{DialysateIn}\left(mmol/l\right)\right] \end{array}$$

###  Assessment of Inflammatory Cytokines Levels 

 The inflammatory markers of interleukin*-*6(IL*-*6), tumor necrosis factor α (TNF-α), IL-2 and mesothelial marker of cancer antigen 125 (CA125) were measured both in the serum and PD effluent. The markers were measured by ELISA using the IBL-International kit (IL-6: cat no. BE53061, IL-2: cat no. BE53021, TNF-α: cat no. BE55001, Ca125: cat no# CA51201, Hamburg, Germany) according to the manufacturer’s protocols. Samples and standards were analyzed in duplicates with a maximum tolerated coefficient of variation (CV) of 20%. We did not report the inter-assay CV, since we assessed all samples from one patient in one run.

 For measurement of serum levels of above markers, 5-mL fasting blood samples were collected at V-1 through V-6 in coagulant-containing vacutainers and the serum was separated and stored at −80°C until all samples were ready for assessment.

 For measurement of PD effluent levels of the above markers, a 5-mL effluent sample was collected after an overnight dwell at V-1 through V-6 and stored at −80°C. The dwell time and volume of the bag were recorded. In order to standardize the levels of the measured marker, the quantity of markers was expressed as appearance rate.^17^

 Appearance Rate = (concentration of cytokine × volume effluent (mL))/ dwell time (min)

###  Assessment of Gene Expression Levels of Fibrosis Markers in PD Effluent - Derived Cells

 Sterile effluent (~2000 mL) was collected from patients after an overnight dwell at V-1 through V-6. Immediately afterwards, the whole effluent was poured into as many as necessary 50-mL sterile falcon tubes and centrifuged at 500 × g and 4°C for 10 minutes and the supernatant was discarded. The precipitated cells from all falcons were added together and washed twice with PBS. The supernatant was discarded and the precipitated cells were placed into 1.5-mL DNase/RNase microtubes containing lysis buffer. The microtubes were then snap-frozen by submerging in liquid nitrogen and stored at -80ºC until all samples were ready for processing.

 We used quantitative real-time polymerase chain reaction (RT-qPCR) analyses to assess the gene expression levels of FSP-1, TGF-*β*, and α-SMA in PD effluent-derived cells.

 For this purpose, total RNA was obtained from frozen samples and isolated using RNeasy Micro Kit (Qiagen, Hilden, Germany) according to the manufacturer’s instructions.

 The total RNA from each sample was reverse-transcribed to cDNA using RevertAid H Minus First Strand cDNA Synthesis Kit (Thermo scientific) for samples with RNA concentrations higher than 0.5 μg, and Quantitec, Whole Transcriptome Kit (Qiagen, Hilden, Germany) for samples with RNA concentrations lower than 0.5 μg, according to the manufacturer’s instructions.

 RT-qPCR was performed to determine the relative gene expression profiles with 2X SYBR Green master mix (Takara) and 10µM of each primer in a total volume of 20 µL. These reactions were performed on StepOnePlus^TM^ Real Time PCR Systems (ABI applied Biosystem). In the next step, the size of product was assessed on 1% agarose gel. To confirm the specific product amplification and elimination of probable contamination, the dissociation curve was analyzed. The relative gene expression values were determined using the 2−ΔΔCt method (where Ct is threshold cycle) of the relative quantification manager software (version 1.2.1, Applied Biosystems) with normalization to the GAPDH. Amplification reactions were performed in duplicate on all samples and for each gene. The primers of each gene are presented in Table 1.

**Table 1:** Sequence of Primers Used in this Study

**Gene**	**Sequence**	**Expected Lengths**
*TGF- β*	Forward:5’-AACATGATCGTGCGCTCTGCAAGTGCAGC-3’ Reverse: 5’-AGGACGGACAGACGTGATA-AGGAA- 3’	200 bp
*S100A4 (FSP-1) *	Forward:5’-AGGGACAACGAGGTGGACTT-3’ Reverse: 5’-CTTCCTGGGCTGCTTATCTGG-3’	105 bp
*ACTA2 ( α -SMA) *	Forward: 5’-AGCGTGGCTATTCCTTCGTTA-3’ 153 bp Reverse: 5’-GCCCATCAGGCAACTCGTAA-3’	153 bp
*GAPDH*	Forward: 5’-AGAAGGCTGGGGCTCATTTG-3’ Reverse: 5’-AGGGGCCATCCACAGTCTTC-3’	258 bp

###  Statistical Analysis 

 Continuous data were shown as mean ± SD. Discontinuous data were shown as primary values or as a frequency. The general linear model was used to calculate repeated measures of ANOVAs for functional and inflammatory parameters and gene expression from baseline at follow-up visits. A p-value less than 0.5 was deemed statistically significant. Analyses were performed using SPSS (Version 16.0, NY-USA).

## Results

 We recruited 10 patients for this study. One of the patients refused to participate in the rest of the study after liposuction and was therefore excluded. All nine patients finished the follow-up. The mean age of subjects was 55.6 years (SD:11.9) and the majority of them were female subjects (66.7%) (Table 2). The mean duration of PD was 77.1 months (min-max: 24–124 months).

**Table 2:** Characteristics of Study Subjects at the Time of Enrollment

**Parameters**	**Mean±SD**	**Pt 1**	**Pt 2**	**Pt 3**	**Pt 4**	**Pt 5**	**Pt 6**	**Pt 7**	**Pt 8**	**Pt 9**
Age (y)	55.6 ± 11.9	47	70	69	43	69	63	44	44	52
Sex (F/M)	6/3	F	F	M	F	M	F	F	F	M
PD duration (mon)	77.1 ± 41.4	81	96	36	25	121	121	124	24	66
BMI (kg/m^2^)	26.9 ± 5.3	19.1	24.6	27.8	26.2	21.6	27.3	36.3	33.7	25.6
24 hours UF (mL)	1216.6 ± 573.4	1300	1600	800	850	1200	300	2000	2000	900
MSCs injected ( × 10^6^)/kg	1.2 ± 0.13	1.2	1.3	1.1	1.3	1.4	1.3	0.9	1.1	1.2
Transport type	—	H	H	HA	HA	HA	H	HA	HA	HA
N of exchange/24 hours	—	5	4	4	5	4	4	4	5	4
FWT (mL)	146.9 ± 56.2	46.5	195.6	201.8	120.4	103.7	121.9	147.1	227.2	157.4
UFSP (mL)	192 ± 158	103.5	204.3	148.2	379.6	-153.7	178	352.9	272.7	242.5
UFT (mL)	338.9 ± 185	150	400	350	500	-50	300	500	500	400
OCG (µL/min/mm Hg)	2.86 ± 2.01	1.5	6.2	8.5	0.58	0	0.95	4.5	5.19	3.18
D/P creatinine	0.75 ± 0.1	0.94	0.82	0.7	0.6	0.69	0.84	0.75	0.65	0.72
D/P urea	079 ± 0.06	0.88	0.82	0.78	0.68	0.71	0.85	0.82	0.79	0.78
D/D0 glucose	0.23 ± 0.05	0.13	0.2	0.24	0.26	0.23	0.19	0.22	0.3	0.26
Na removal (mmol)	25.5 ± 21.9	13.4	28.2	20.1	52	-22.7	23.5	48	36	30.8

F, female; M, male; PD, Peritoneal dialysis; BMI, Body mass index; H, high; HA, high average; FWT, free water transport; UFSP, ultra filtration- small pore; UFT, ultrafiltration total; OCG, Osmotic conductance to glucose; MSCs, Mesenchymal stem cells. D/P creatinine, dialysate/plasma ratio of creatinine; Dt/D0 glucose, dialysate /initial dialysate ratio of glucose. Transport type based on D/P creatinine: > 0.80 = High, 0.65-0.8 = High average, 0.55-0.64 = Low average, < 0.55 = low. Data are presented as means ± SD.

###  Mild Improvement in Peritoneal Membrane Function after AD-MSCs Injection

**Figure 1:**
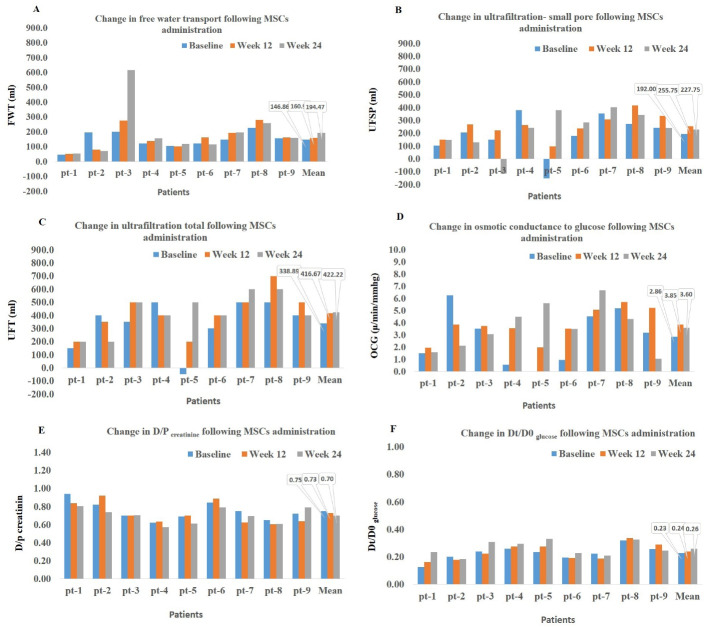
Time Trends of Changes in Functional and Permeability Characteristics of Peritoneal Membrane before (Baseline) and 12 and 24 Weeks after Mesenchymal Stem Cell Injection. D/P creatinine, dialysate/plasma ratio of creatinine; Dt/D0 glucose, dialysate /initial dialysate ratio of glucose

 We detected a 32% increase in FWT (*P* = 0.4) (Figure 1A), 18% increase in UFSP (*P* = 0.05) (Figure 1B), and 25% increase in UFT (*P* = 0.2) (baseline vs. week 24, Figure 1C). We also measured the OCG which is a measure to quantify the ability of PM to generate UF in response to the osmotic agent of glucose. OCG increased by 25% from baseline (2.86 µL/min/mm Hg) to 24 weeks after treatment (3.6 µL/min/mm Hg) (*P* = 0.4) (Figure 1D). The D/P creatinine and Dt/D0 glucose, as the parameters of solute transport, were improved after treatment with AD-MSCs, although only the decrease in D/P creatinine reached the significant level (*P* = 0.02, baseline vs. week 24) (Figure 1E-F).

###  No Significant Changes in Systemic Levels of Inflammatory Markers after AD-MSCs Injection

**Figure 2:**
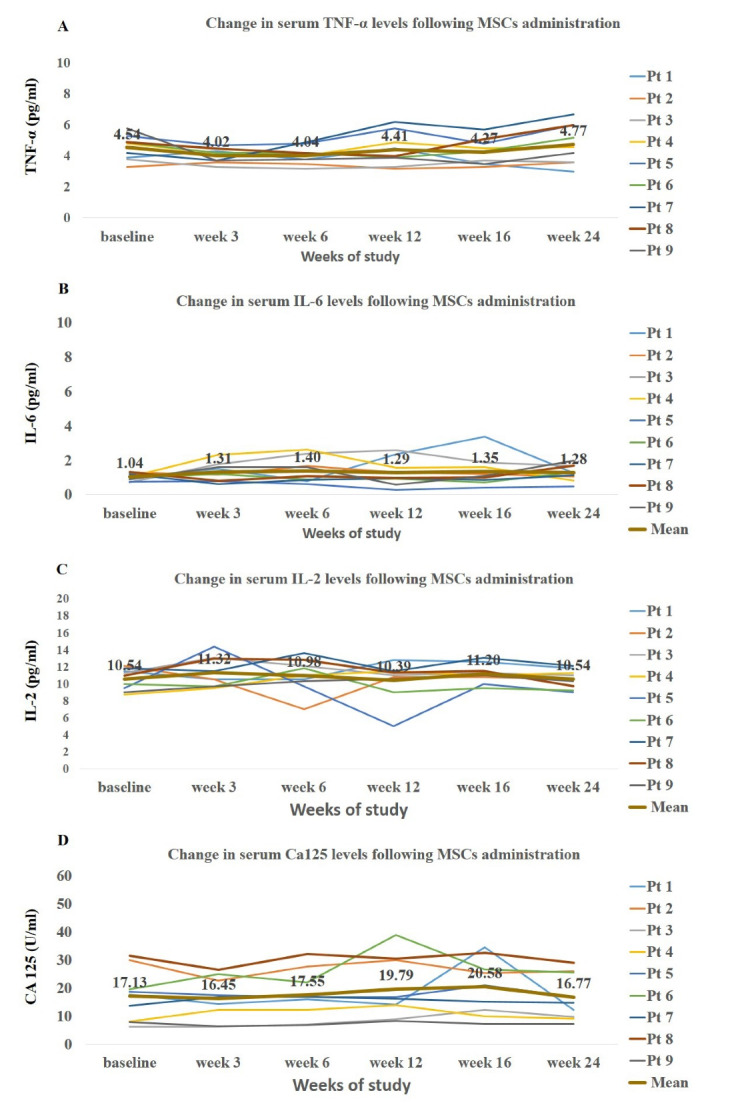
Time Trends of Changes in Systemic Levels of Inflammatory Markers and Mesothelia Marker of CA125 in Patients at Baseline and after Mesenchymal Stem Cell Injection

 There were no significant changes in the mean values of measured markers following treatment with AD- MSCs (Figure 2A-D). The mesothelial marker of CA125 showed a slight increase from baseline to week 16 (17.13 vs. 20.58 U/mL), and then, similar to the other measured markers, diminished by the end of the study (16.77 U/mL). As shown in Figure 2, there was a large inter-individual variability among the included patients.

###  No Significant Changes in Peritoneal Fluid Levels of Inflammatory Markers after AD-MSCs Injection

**Figure 3:**
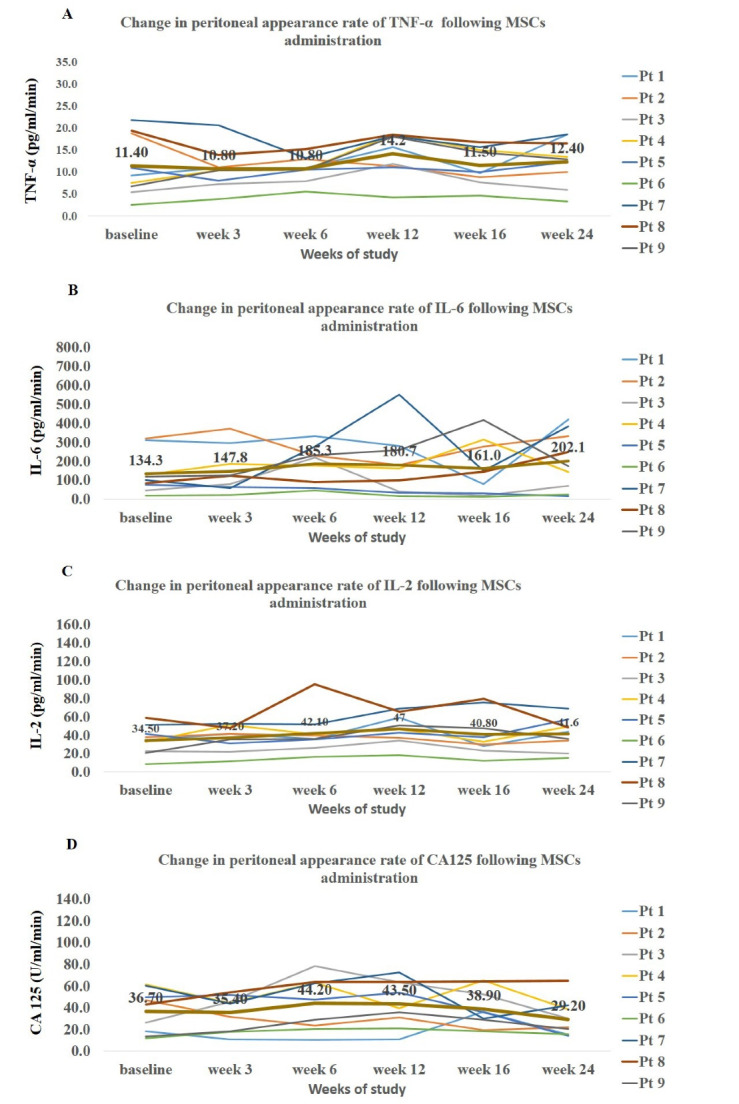
Time Trends of Changes in Peritoneal Levels of Inflammatory Markers and Mesothelia Marker of CA125 in Patients at Baseline and after Mesenchymal Stem Cell Injection

 The quantity of measured markers in effluent was expressed as appearance rate. There were no significant changes in peritoneal appearance rate of TNF-α, IL-6, IL-2 and CA125 at follow-up visits. Notably, the time pattern of these markers showed a slight non-significant increase up to week 12 (Figure 3) followed by gradual decrease up to the end of the study.

###  Mild Changes in Gene Expression Levels of Fibrosis Markers in Effluent-Derived Cells after AD-MSCs Injection

**Figure 4:**
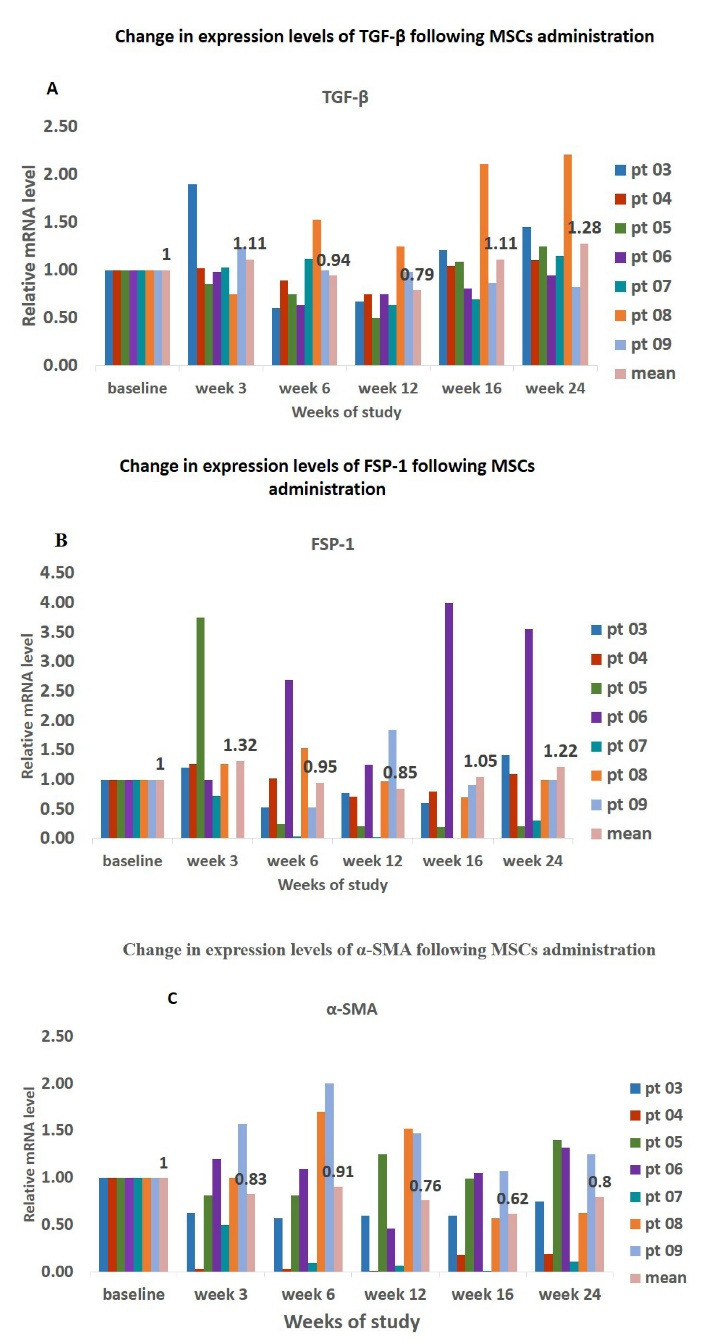
Time Trends of Changes in Gene Expression Levels of Fibrosis Markers in Effluent-derived Cells at Baseline and after Mesenchymal Stem Cell Injection.

 There was a slight non-significant reduction in expression levels of TGF-β and FSP-1 from baseline up to 12 weeks (visit 4) after AD-MSCs treatment in PD patients (TGF-β: 1 vs. 0.79, FSP-1: 1 vs. 1.22); however, afterwards, the expression levels of TGF-β increased and reached 1.28 (Figure 4). A similar scenario was observed for α-SMA where the lowest level of expression was found at 16 weeks after treatment with AD-MSCs (1 vs. 0.62).

## Discussion

 Previously, we have reported that systematic administration of autologous AD-MSCs in CAPD patients suffering from UFF is safe and tolerated. Here, we searched for potential therapeutic effects of autologous MSC and found that one-time systemic therapy with AD-MSC had small beneficial effects on fluid and solutes kinetics, as demonstrated by improvement in FWT, UFSP, OGC and D/P creatinine. We also found slight transient diminution in gene expression levels of TGF-β, FSP-1 and α-SMA in PD effluent-derived cells. To the best of our knowledge, this is the first clinical trial that reports the effects of systemic therapy with autologous AD-MSCs on different parameters of PM.

 Overtime use of conventional PD fluids alter both the anatomical and functional integrity of the PM.^18^ This progressive damage to PM is evidenced by the gradual increase in low molecular weight solute transport and diminished UF capacity and probability of UFF.^19^ Based on the 3-pore theory of transport, the transcapillary UF occurs through both small interendothelial pores (radii of about 40 Å) and intra-endothelial water channel aquaporin-1 that acts as the ultrasmall pore.^18^ The transcellular membrane protein of aquaporin-1 allows only water transport, but not solutes, and therefore induces FWT. While the small interendothelial pores are involved in transport of both low molecular weight solute and water, recent evidence suggests that UF is more complex and a combination of submesothelial fibrosis, angiogenesis and augmented vessel permeability are the key elements of UF dysfunction.^20^

 In this study, we used UNI-PET to examine the course of changes in PM function. To our knowledge, very few studies have used this test, perhaps because of its complex and time-consuming nature. Using this test provided us with an extended and more accurate evaluation of UF capacity and allowed us to simultaneously assess the changes in UF produced from different parts of the PM (FWT, UFSP, UFT), solute transport (D /P Creatinine, Dt/D0 glucose) as well as OCG, which is an indirect indicator of fibrosis in peritoneal interstitium.

 Temporal assessment of peritoneal function parameters following MSCs infusion showed slight improvement in all indices of PM transport. Accordingly, several *in-vivo* studies involving animal models of human PD demonstrated the promising ameliorating effects of stem cell therapy on different parameters of peritoneal function as demonstrated by increase in UF and D/D0 glucose and decrease in glucose mass transfer and D/P creatinine or D/P BUN.^21-25^ Sekiguchi et al, in a chlorhexidine gluconate (CG)-induced PF mouse model, showed the accumulation and maintenance of intraperitoneally transplanted bone marrow-derived cells in the submesothelium for 3 weeks after discontinuation of peritoneal injury and attributed the improvement of peritoneal function to the contribution of stem cells in the process of mesothelial remodeling.^26^ On the contrary, Ueno et al showed the prolonged anti-inflammatory and anti-fibrotic effects of peritoneally injected bone marrow-derived MSCs in the peritoneum beyond the MSC disappearance in a CG-induced PF rat model; a finding that pointed to paracrine activities of MSCs independent of their transdifferentiation into functional peritoneal mesothelial cells.^22^ Accordingly, injected MSCs-condition medium produced similar effects to injected MSCs with respect to reduction of adhesion formation in a rat model of PF.^27^ We do not know the precise mechanism by which MSCs might have acted in our study because of the complexity of the chronic fibrosis process as occurs in patients under long-term dialysis. However, it is more likely that infused MSCs exerted their effect via inflammatory modulation of local environment and mesothelium protection rather than differentiation and structural support of the injured tissue.

 It has been shown that inflammation plays an important role in the injury of PM and small solute transport across it.^28,29^ A persistent systemic and intraperitoneal inflammatory state is reported in patients under long-term dialysis.^28-30^ To verify whether injected MSCs affected the inflammatory status of CAPD patients, we measured both the systemic and intraperitoneal levels of cytokines including IL-6, TNF-α and IL-2 and also CA125 (as a measure of mass of mesothelial cells) before and after MSCs treatment. In order to standardize the intraperitoneal levels of measured factors, we used the peritoneal appearance rate instead of reporting the absolute levels.^16^ While there was a slight non-significant increase in both the systemic and local levels of CA125, especially in month 3, overall, we were not able to correlate the peritoneal function improvement with local and systemic cytokine variation profiles. Our published systematic review on experimental studies of PF also showed great variation in regard to cytokine profile changes following MSC treatment.^12,31^ The lack of a significant change in inflammatory metrics following MSCs injection might be related to diffuse tissue injury in our patients. On the other hand, the slight increase in CA125 following MSCs might suggest that MSCs can increase the mesothelial mass, perhaps via attenuation of mesothelial degradation and promotion of endogenous mesothelial repair processes. Accordingly, animal models of PF showed a significant increase in rate of mesothelial recovery following MSCS treatment; a finding that was correlated with improved peritoneal function.^26,27,32^

 Studies have provided direct evidence for the principal role of TGF-β, both as an inducer and regulator in PF development irrespective of etiology.^22,33,34^ TGF-β secretion is central for MMT program in which, in a complex process, the mesothelial cells phenotype is converted into a mesenchymal one with loss of polarization and gaining the fibroblastic shape.^35^ While the epithelial features are lost, mesothelial cells gain expression of molecules related to MMT, such as α-SMA and FSP-1^4^ and eventually become a main source of myofibroblasts. In this regard, we measured the expression of mesenchymal markers of α-SMA and FSP-1 and also TGF-β, in PD effluent-derived cells and observed that our treatment produced a slight inhibition in the expression of these markers. This inhibition was more prominent in month 3 (visit 4) and gradually vanished by the end of the study (visit 6). Interestingly, the peak of decline in expression of these profibrotic genes corresponded to the time of optimal improvement in peritoneal UF and function. In an *in-vitro* study, Wei and colleagues showed that treatment of renal tubular cells with MSC inhibited the EMT process through decreasing TGF-β1.^36^ Bastug and colleagues^21^ in a rat model of chronic PD showed the positive effects of MSCs on UFF; a finding that was associated with decreased levels of TGF-β 20. Ueno et al, in a CG-stimulated PF rat model, demonstrated that intraperitoneal injection of MSCs suppressed peritoneal expression of α-SMA, FSP-1 and TGF-β; a finding that was associated with improved peritoneal function.^22^ It is shown that an expanded fibrotic interstitium restricts water transport, as it produces an extra barrier between capillaries and the dialysis fluid. It also decreases the dissemination of glucose around peritoneal capillaries, lowering the osmotic gradient across the capillaries.^37,38^ Taken together, one might postulate that MSCs contributed to ameliorating the peritoneal function in our study by suppressing the TGF-β signaling which in turn attenuated the fibrosis state in the peritoneal interstitium manifested by the small decrease in effluent cell-derived gene expression of α-SMA and FSP-1. Given the high variability of immunological data and the limited number of patients included in our study, it is reasonable to state that we would have been able to detect significant results, if the effects had been very strong. Clearly, it was not the case in our study and that might explain why we saw only small changes. However, whether these effects were produced via paracrine activity or by integrating the mesenchymal cells into local tissue needs to be answered.

 We have to mention that obtaining peritoneal tissue biopsy in PD patients is a very high risk procedure as the PM of these patients is highly inflamed and damaged and susceptible to infection. To decrease the risk, in this study, we assessed the changes in expression levels of fibrotic markers following MSCs treatment in effluent derived cells instead of mesothelial cells.

 Certainly our study has some limitations. First, since it was not a randomized controlled trial, the changes cannot be entirely attributed to MSC treatment. Second, because of ethical issue, we could not track homing of MSCs to the peritoneum as the injected MSCs were not labeled. Lastly, because of ethical issues, we could not assess the changes in peritoneal tissues which indeed could have given us a more accurate picture of histological changes following MSCs treatment. Despite these limitations, our data provides justification for further clinical testing.

 In conclusion, the overtime loss of peritoneal UF capacity calls for discovery of interventions that preserve or restore the peritoneal function. Here, we assessed the effects of AD- MSCs transplantation on peritoneal function and inflammation. Our results suggest that MSCs have potent therapeutic effects for peritoneal damage and fibrosis. However, many issues including appropriate administration protocol regarding sources and timing, appropriate number of MSCs to be administered, and need for re-administration in order to maintain the effects should be clarified before we could practically employ MSCs in these patients. Therefore, our results should be regarded as hypothesis suggestion and will need confirmation in future studies.
